# Laparoscopic approach to pheochromocytoma in pregnancy: case report

**DOI:** 10.1590/S1677-5538.IBJU.2017.0540

**Published:** 2018

**Authors:** Felipe de Almeida e Paula, Ravisio Israel dos Santos, Odivaldo Antonio Ferruzzi, Rafael Osti de Melo, Mariana Takaku

**Affiliations:** 1 Hospital Regional do Câncer de Presidente Prudente, Presidente Prudente, SP, Brasil; 2 Santa Casa de Misericórdia de Presidente Prudente, Presidente Prudente, SP, Brasil; 3 Faculdade de Medicina de Presidente Prudente, Universidade do Oeste Paulista, Presidente Prudente, SP, Brasil

**Keywords:** Pheochromocytoma, Pregnancy, Laparoscopy

## Abstract

A 32-year-old 22-week pregnant hypertensive woman with sporadic episodes of headaches, sweating, and facial flushing was diagnosed with pheochromocytoma through biochemical and imaging tests. Perioperative management included a multidisciplinary approach, symptom stabilization with α blockade followed by β blockade, and tumor resection by laparoscopic adrenalectomy at 24 weeks gestation. The diagnosis was confirmed by histopathological examination and immuno-histochemistry tests. The decision for surgical removal of the tumor was based on maternal symptoms, tumor size, gestational age, the possibility of doing a laparos-copy, and the expertise of the surgical team.

## INTRODUCTION

Pheochromocytoma (PCC) is a neuroendocrine tumor derived from chromaffin cells and located within the adrenal medulla in approximately 90% of cases (1). Although extraordinarily rare during pregnancy with a reported incidence less than 0.2 per 10,000 pregnancies, PCC should be considered when severe hypertension occurs before 20 weeks gestation or when hypertension is associated with disease symptoms such as headaches, palpitations, and diaphoresis (2). PCC in gestation, when untreated, raises maternal and fetal mortality to up to 50% (3, 4). However, early diagnosis and appropriate treatment reduce maternal mortality to less than 5% and fetal mortality to below 15% (3, 5). The diagnosis can be established by detecting elevated levels of catecholamines and their metabolites in plasma or urine. Ultrasound and magnetic resonance imaging (MRI) are the preferred imaging modalities for tumor localization during pregnancy (6). Surgery is the definitive treatment for PCC after management of hypertension with **α** blockade, but the timing of tumor removal depends on the gestational age, the severity of maternal symptoms, and the risks associated with pregnancy termination (4).

## CASE PRESENTATION

**Figure 1:**
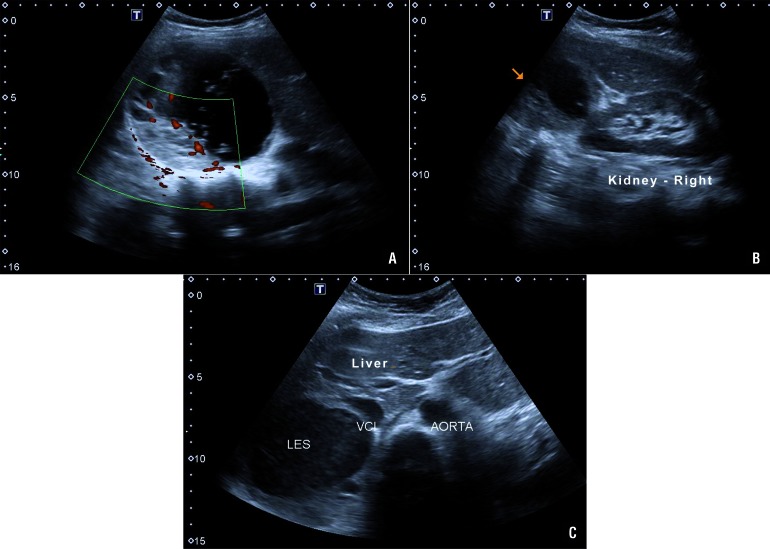
A) Ultrasound showing right adrenal tumor with more than 10 centimeters, with mixed cystic-solid components and doppler imaging confirm the presence of vascular flow; B) Arrow indicating the great mass between the liver (Liver) and the right kidney (Kidney - right); C) pheochromocytoma (LES) by compressing and pushing the inferior vena cava (VCI).

A 32-year-old Caucasian woman at 22 weeks of gestation with singleton live fetus presented 5 weeks prior with sporadic episodes of headaches, facial flushing, and sweating. The obstetric ultrasound showed a healthy fetus. Her first pregnancy four years earlier was complicated by severe preeclampsia at 34 weeks gestation for which she was induced and delivered by emergency cesarean section. Since then, she was treated with methyldopa 500 mg/day, but her blood pressure was not monitored and she had no other complaints or complications. She was not on any regular medications, was not a smoker or an alcohol user, and there was no history of illicit drug use nor family history of multiple endocrine neoplasia (MEN). Due to palpitations, nine months earlier she underwent a cardiac stress test, which detected sustained supraventricular paroxysmal tachycardia, followed by an electrophysiology study that failed to reveal any disorder or dysfunction. The echocardiogram was also normal. Symptoms of head-aches, facial flushing, and sweating started at 17 weeks gestation. Routine laboratory tests re-vealed a fasting glucose of 92 mg/dL and serum creatinine of 0.5 mg/dL. Complete blood count, sodium, potassium, calcium, phosphorus, AST, ALT, total cholesterol and fractions, TSH, free T4, urinary sediment levels, serum aldosterone, calcitonin, parathyroid hormone (PTH), and cor-tisol were all normal. However, metanephrine and normetanephrine on 24 hours urine collection were 6873.2 μg/24 h (reference value <280 μg/24 hours) and 6299.2 μg/24 hours (reference value <732 μg/24 hours), respectively. Ultrasonography of the abdomen revealed a 10.1 × 9.5 cm mass in the right adrenal gland consistent with PCC (Figure-1). Blood pressure was controlled with prazosin 2 mg/day for 10 days followed by propranolol 20 mg/day for four days, when a consultation by a multidisciplinary team, including surgeons, anesthesiologist, obstetrician, and endocrinologist, was held to determine the most appropriate management. The surgical approach selected was laparoscopic transperitoneal right adrenalectomy due to the considerable experience of the surgical team with the technique, the patient's significant symptoms, and the gestational age (second trimester, 24 weeks gestation at the time of surgery). The patient was placed in a modified flank position, 45 degrees back from vertical. Three ports were used in triangulation, and an additional fourth port for the liver retraction. The patient received combined epidural and general anesthesia with an epidural catheter placed for postoperative analgesia and was continuously monitored with a cardioscope, pulse oximetry, in-vasive arterial pressure monitoring from the radial artery, and capnography. Serial arterial blood gases and glucose were monitored to prevent maternal acidosis and hypoglycemia. Central venous access was accomplished via her right jugular vein for administration of liquids and vasoactive agents. General anesthesia was maintained with continuous remifentanil and propofol infusion. Special consideration was taken with pneumoperitoneum pressure, which was maintained below 12 mmHg for nearly the entire duration of surgery and was only raised to 15 mmHg for a few minutes during vessel dissection to prevent a reduction in uteroplacental blood flow. During dissection of the adrenal vessels, a sodium nitroprusside infusion (0.8 μg/kg/min for approximately 7 min.) and intermittent boluses of esmolol 10 mg IV (total 20 mg) were used to control mean arterial pressure (MAP reduced from 136 mmHg to 83 mmHg) and heart rate (lowered from 133 bpm to 86 bpm). An ultrasonic dissector - Harmonic scalpel™ (Ethicon Endo Surgery INC - Johnson & Johnson, NJ, USA) was used for adrenal gland dissection. Blood loss was less than 50 mL. The patient remained normotensive for the remainder of the surgery and postoperatively. The operative time was 120 min and no complications were recorded. The patient was extubated immediately after surgery with good pain control, and the epidural catheter was removed on the same day of surgery. Obstetric ultrasound performed soon after anesthesia recovery revealed no abnormalities. Antihypertensive therapy was not required postoperatively or during the remainder of pregnancy. The patient was discharged on postoperative day four with no symptoms and normal blood pressure.

Histopathological examination and immu-nohistochemistry tests (positive chromogranin A and synaptophysin staining) confirmed the diagnosis of PCC.

Five weeks after surgery, while remaining normotensive without antihypertensive drug therapy, the patient was admitted due to premature rupture of membranes, and one day later suffered placental abruption with severe bleeding. She underwent emergency cesarean section but the newborn, who was born alive, died prematurely within 48 hours.

## DISCUSSION

PCC in pregnancy can cause fatal hypertensive crisis that can be triggered by vaginal delivery, general anesthesia, the physical effects of the enlarging uterus, uterine contractions, or fetal movements (2), and may impose a serious risk to the fetus because extreme vasoconstriction in the uteroplacental circulation may result in intrauterine hypoxia and premature placental abruption (5). Undiagnosed and/or untreated PCC carry a risk of mortality for both mother and fetus as high as 58% (4, 6).

Symptoms may occur for the first time in pregnancy due to increased vascularity of the tumor and/or mechanical factors such as pressure from the enlarging uterus or fetal movements, which can stimulate catecholamine secretion (7, 8). These signs and symptoms include hypertension (98% of cases), orthostatic hypotension, palpitations, headaches, sweating, anxiety attacks, facial flushing, and chest pain (5, 6). However, in pregnant women, the disease can be mistaken for other causes of hypertension, especially specific hypertensive disorders of pregnancy such as pre-eclampsia (3). In the current case, preeclampsia in the previous pregnancy may have been the first manifestation of PCC, but this hypothesis was not investigated at the time.

Detecting elevated levels of catecholamines and their metabolites in plasma and urine establishes the diagnosis of PCC. Once a diagnosis has been confirmed biochemically, ultrasonography and MRI are the preferred modalities of tumor localization for the safety of the fetus (2, 4, 5).

The success of treatment will depend on the appropriate preoperative medical management, which should always be started with α-adrenergic blockade (phenoxybenzamine or prazosin) for adequate control of blood pressure (9). Pre-surgical preparation with this class of drugs is one of the main reasons why surgical mortality has decreased over the last 30 years to <3% (5). Even though there is no consensus regarding the optimal duration of medical pretreatment, it should be given for 10-14 days or until a stable hemodynamic condition is achieved. In case of tachyarrhythmias, β-adrenergic blockade should only be started after some days of appropriate α-adrenergic blockade (2, 4, 7). A collaborative multidisciplinary team, including surgeons, anesthesiologist, obstetrician, and endocrinologist, is decisive for case management (10). Surgical resection of the tumor is the definitive treatment for PCC, which can be achieved either by open, laparoscopic, or robotic approaches (1, 6, 8). Laparoscopic adrenalectomy is the surgical approach of first choice if tumor size is <7 cm (4, 10, 11); this is a safe procedure with a complication rate <8% (5, 9). Although the posterior retroperitoneoscopic approach seems to be a good alternative for adrenalectomy than laparoscopic transperitoneal approach, avoiding the need to enter the peritoneal cavity, there is no evidence that supports the superiority of one over the other, both showing similar low morbidity and mortality (11). The choice of surgical approach must be based mainly on the expertise of the surgical team and the greater maternal and fetal safety. In this case, laparoscopic transperitoneal approach was chosen, considering the greater experience with this technique by the surgical team, as well as the unfeasible prone position of pregnant patient to perform a posterior retroperitoneoscopic approach.

Timing of tumor excision in pregnant women will depend on the gestational age at which di-agnosis is made, fetal development, and the severity of maternal symptoms. Surgery should be avoided in the first trimester — as organogenesis is incomplete and miscarriage is highly likely — as well as in the third trimester — because by then the enlarging uterus precludes adequate access and visualization of the abdomen (12). Thus, the second trimester is recommended as the ideal time for surgical intervention. Surgical removal is recommended before 24 weeks of gestation (3, 5, 7, 8). However, after 24 weeks of gestation, the patient can be treated with the appropriate α-adrenergic blockade until the fetus is viable, when the tumor can be removed after an elective cesarean section (4, 7, 8). In the current case, following a multidisciplinary consultation and with the patient's consent, and having been treated with prazosin for 10 days, she underwent laparoscopic adrenalectomy at 24 weeks gestation.

Pregnancy was previously a contraindication to laparoscopy because of concerns regarding the potential effect of carbon dioxide on the developing fetus and the safe entry into the abdominal cavity due to the displacement of anatomy by the gravid uterus. Additionally, increased abdominal tension by the pneumoperitoneum could reduce the vena cava and the uteroplacental blood flows (1, 8). Nevertheless, previous concerns surrounding the potential adverse hemodynamic effects associated with pneumoperitoneum were unjustified, because these changes were no different when compared to open adrenalectomy (2). Moreover, catecholamine concentrations increase to a lesser extent with laparoscopic than with open adrenalectomy (2). Thus, laparoscopy is currently the safest adrenalectomy approach even for this high-risk population. Laparoscopy is a less invasive procedure that results in faster functional recovery, shorter hospital stay, and early ambulation, reducing the risk of thromboembolism and the need for opioid painkillers, which can affect fetal development. In addition, return of gastrointestinal function is also more rapid than with an open procedure (10, 11). Small incisions decrease the potential for herniation with the increased pressure of the gravid uterus and, in addition, laparoscopy offers enhanced visualization without manipulating the uterus, resulting in reduced risk of preterm labor (1, 2, 5, 8). These benefits combined with the expertise of the surgical team and a multidisciplinary approach make laparoscopy a safe, effective, and minimally invasive option for adrenalectomy for PCC in pregnancy (1).

Importantly, perioperative management includes treatment of both mother and fetus (4). In the current case, management was effective, without intra or postoperative complications, resulting in complete resolution of the disease. Despite the perinatal fetal demise, maternal safety was pre-served and delaying surgical resection could have increased the risk of catastrophic complications in late pregnancy.

## CONCLUSIONS

PCC is a rare but important cause of hypertension in pregnancy. It should be included and investigated as a differential diagnosis in cases of labile hypertension in pregnant women with or without associated symptoms, because inadequate treatment considerably increases maternal and fetal mortality. Management of PCC during pregnancy should be discussed on a case by case basis by a multidisciplinary team. Surgical resection is recommended during the second trimester of pregnancy. Considering its benefits and the expertise of the surgical team, laparoscopic surgery is the favored technique.
